# Supplementary material for: Identification of photoperiod-induced specific miRNAs in the adrenal glands of Sunite sheep (Ovis aries)
Source: Front Vet Sci. 2022 Jul 22;9:888207. doi: 10.3389/fvets.2022.888207 (PMC9354845; doi:10.3389/fvets.2022.888207)
Supplement: Supplementary file 1 [file Data_Sheet_1.ZIP › Supplementary file/miRDeep2 file.zip!/miRDeep2 file/Know_Structure/SPLP42A1/oar-mir-541.pdf]

miRBase precursor : oar-mir-541
Total read count : 325
oar-miR-541-5p read count : 317
oar-miR-541-3p read count : 7
remaining reads : 1

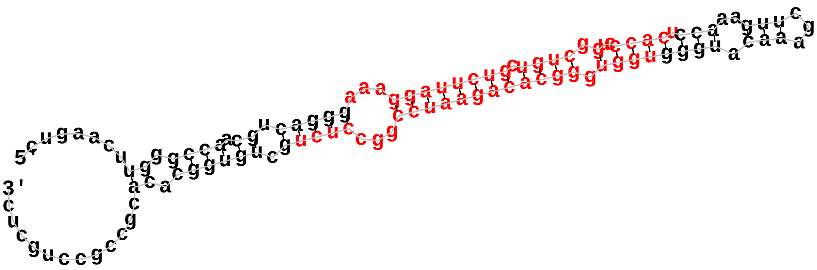

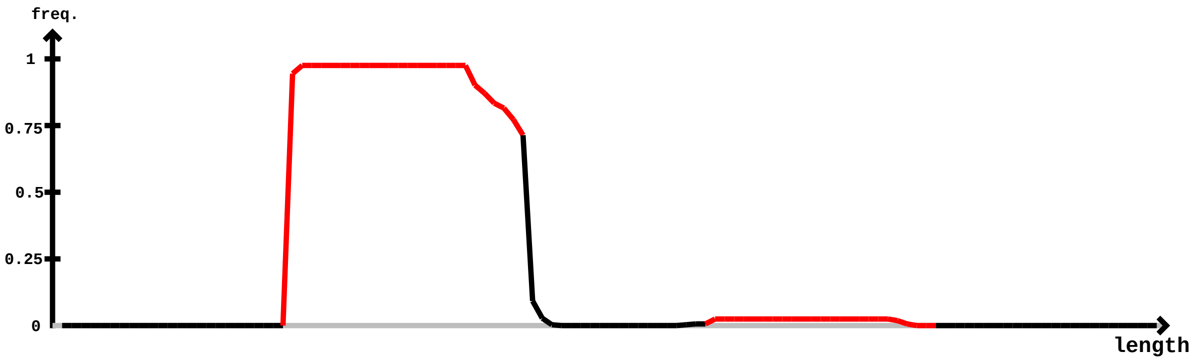


```
                                                                          oar-miR-541-3p
                         oar-miR-541-5p
5'- cugaacuugggccaacgucagggaaaggauucugcugucggucccacuccaaaguucgaaacauggguggugggcacagaauccggccucugcuguggcacacgccgccugcuc   -3'    exp
    ........((((..(((.(((((...((((((((.((((.(..((((.(((..(((...))).)))))))).))))))))))))...))))).)))(((....)))))))....    reads    mm    sample
    .......................aaaggauucugcugucggu........................................................................    24    0    seq
    .......................aaaggauucugcugucgguc.......................................................................    10    0    seq
    .......................aaaggauucugcugucggucc......................................................................    12    0    seq
    .......................aaaggauucugcugucgguccc.....................................................................    6    0    seq
    .......................aaaggauucugcugucggucccа....................................................................    14    0    seq
    .......................aaaggauucugcugucggucccac...................................................................    17    0    seq
    .......................aaaggauucugcugucggucccacu..................................................................    195    0    seq
    .......................aaaggauucugcugucggucccacuc.................................................................    20    0    seq
    .......................aaaggauucugcugucggucccacucc................................................................    8    0    seq
    .......................aaaggauucugcugucggucccacucca...............................................................    1    0    seq
    ........................aaggauucugcugucggucccac...................................................................    2    0    seq
    ........................aaggauucugcugucggucccacu..................................................................    7    0    seq
    ........................aaggauucugcugucggucccacuc.................................................................    1    0    seq
    ................................................................ggguggugggcacagaauccggcc..........................    1    0    seq
    .................................................................gguggugggcacagaauccggc...........................    1    0    seq
    ...................................................................uggugggcacagaauccgg............................    2    0    seq
    ...................................................................uggugggcacagaauccggc...........................    3    0    seq
    ...................................................................uggugggcacagaauccggcc..........................    1    0    seq
```
